# Compensatory Masseteric Bulging: A Novel Observation and Its Implications for Botulinum Neurotoxin Injection Techniques

**DOI:** 10.1111/jocd.70090

**Published:** 2025-04-07

**Authors:** Kyu‐Ho Yi, Jovian Wan

**Affiliations:** ^1^ You and I Clinic Seoul Korea; ^2^ Division in Anatomy and Developmental Biology, Department of Oral Biology, Human Identification Research Institute, BK21 FOUR Project Yonsei University College of Dentistry Seoul Korea; ^3^ Medical Research Inc. Wonju South Korea

## Abstract

**Aims:**

Compensatory masseteric bulging, a newly identified complication, arises from repeated botulinum neurotoxin injections targeting the lower mid‐masseter in East Asians. This phenomenon occurs when untreated upper muscle layers hypertrophy to compensate for weakened lower regions, disrupting facial symmetry. Traditional injection strategies, focused on the lower muscle bulk, overlook the masseter's complex three‐layered anatomy (superficial, middle, deep), increasing asymmetry risks.

**Methods:**

To prevent compensatory bulging, a retrograde, layered injection technique is proposed, distributing botulinum neurotoxin evenly across the upper, middle, and lower masseter. Ultrasound guidance enhances precision, ensuring toxin delivery to targeted layers while avoiding diffusion into adjacent muscles (e.g., risorius). Personalized dosing, adjusted for muscle thickness, activity, and treatment history, minimizes localized over‐atrophy.

**Results:**

A 34‐year‐old female developed upper masseter bulging after four lower mid‐masseter botulinum toxin sessions over two years. Ultrasound revealed upper hypoechoic hypertrophy (12 mm thickness) contrasting with lower hyperechoic atrophy (5 mm). Injecting 50 units of LetibotulinumtoxinA into the upper masseter reduced hypertrophy (8 mm post‐treatment), restoring facial symmetry.

**Discussion:**

Compensatory bulging underscores the need for holistic treatment addressing the entire muscle. Layered injections, guided by ultrasound and tailored dosing, mitigate asymmetry risks. Clinicians must adopt comprehensive strategies, integrating anatomical insights and advanced imaging, to optimize aesthetic outcomes in masseter hypertrophy management.

## 1 Introduction

Masseter muscle hypertrophy is a prevalent aesthetic concern, particularly in individuals of East Asian descent, where a broad, square‐shaped lower face is often considered less desirable. This condition, characterized by the enlargement of the masseter muscles, contributes to a wider and more angular facial appearance. Botulinum neurotoxin injections have become a widely accepted nonsurgical solution for reducing the size of these muscles, allowing for a softer and more streamlined facial contour [1, 2, 3, 4]. The effectiveness of botulinum neurotoxin in aesthetic medicine is well established, particularly in its ability to inhibit acetylcholine release at the neuromuscular junction, thereby reducing muscle activity and leading to gradual atrophy [5]. However, the masseter muscle's complexity, with its three distinct layers—superficial, middle, and deep—poses treatment challenges. These layers, with their varied origins and insertions along the zygomatic arch and mandible, require precise targeting to achieve desired outcomes without adverse effects. Traditionally, botulinum neurotoxin injections for masseter hypertrophy have focused on the lower mid‐part of the muscle, where the bulk is most prominent [4]. While this approach effectively reduces muscle mass in the targeted area, repeated treatments can lead to unintended consequences.

A novel phenomenon, referred to as compensatory masseteric bulging, has been identified and described in this article. This condition occurs when the upper portion of the masseter muscle undergoes hypertrophy as a compensatory response to the repeated weakening of the lower mid‐part of the muscle. This phenomenon has not been previously documented in the literature, and it presents a unique challenge in the management of masseter hypertrophy. Compensatory masseteric bulging can lead to an uneven and asymmetrical facial appearance, counteracting the intended aesthetic benefits of the treatment.

The purpose of this article is to introduce and elaborate on the concept of compensatory masseteric bulging, discuss its anatomical underpinnings, and propose innovative strategies for preventing and managing this complication in clinical practice. By providing a detailed exploration of the anatomy of the masseter muscle and the dynamics of botulinum neurotoxin diffusion, we aim to enhance the precision and efficacy of treatments aimed at reducing masseter hypertrophy.

## 2 Innovative Ideas

Understanding and addressing compensatory masseteric bulging requires a re‐examination of the anatomy of the masseter muscle and the mechanisms by which botulinum neurotoxin affects it. The masseter muscle, which plays a crucial role in mastication, is a thick, quadrilateral muscle with three distinct layers: superficial, middle, and deep. The superficial layer is the most prominent and contributes significantly to the muscle's bulk, while the middle and deep layers, located beneath, provide additional structural support and function [6, 7].

Botulinum neurotoxin works by inhibiting the release of acetylcholine at the neuromuscular junction, leading to temporary paralysis of targeted muscle fibers [4, 8]. Over time, this results in a reduction of muscle bulk due to decreased muscle activity. When injections are repeatedly concentrated in the lower mid‐part of the masseter, the treated area becomes progressively weaker. However, the untreated upper portion of the muscle may respond to this imbalance by undergoing hypertrophy, leading to compensatory masseteric bulging.

To mitigate this risk, it is essential to revise current injection practices by adopting a more comprehensive approach. First, injections should be evenly distributed across the entire masseter muscle, including the upper, middle, and lower sections. This balanced approach ensures that no single area of the muscle is left untreated, thereby reducing the risk of compensatory hypertrophy. Second, adopting a layered injection technique, where the botulinum neurotoxin is administered retrogradely across the muscle's superficial, middle, and deep layers, can help ensure even distribution of the toxin. This method minimizes localized weakening and subsequent compensatory hypertrophy in untreated areas.

Precise dosage management is equally critical in mitigating the development of compensatory masseteric bulging. The patient's individual muscle response to botulinum neurotoxin varies according to factors such as muscle thickness, level of activity, and prior treatment history. Thus, tailoring the dosage to the specific needs of each patient, rather than adhering to a standardized regimen, is essential. Excessive dosing in a single area may precipitate rapid atrophy, which could exacerbate compensatory hypertrophy in other regions of the muscle. Regular monitoring of patient outcomes, with dosage adjustments as necessary, can significantly reduce these risks.

Furthermore, the incorporation of ultrasound guidance during botulinum neurotoxin injections can enhance the precision and efficacy of treatment. Ultrasound imaging allows for real‐time visualization of the masseter's anatomical layers, facilitating accurate placement of the neurotoxin. This advanced technique not only ensures that the toxin is delivered precisely to the intended muscle fibers but also reduces the risk of inadvertent diffusion to adjacent muscles, such as the risorius, which could result in undesirable complications like asymmetrical facial expressions.

## 3 Case Report

A 34‐year‐old female patient presented with concerns regarding a broad lower face, attributed to hypertrophy of the masseter muscles. Over the past 2 years, she has received botulinum neurotoxin (letibotulinum toxin A) injections targeting the lower mid‐part of the masseter muscle at regular intervals (6 month interval of 4 sessions). Initially, the treatments were effective, resulting in a noticeable reduction in facial width and a more streamlined appearance. However, the patient recently began to observe a prominent bulging in the upper portion of her masseter muscle, which led to an asymmetrical facial contour.

**FIGURE 1:**
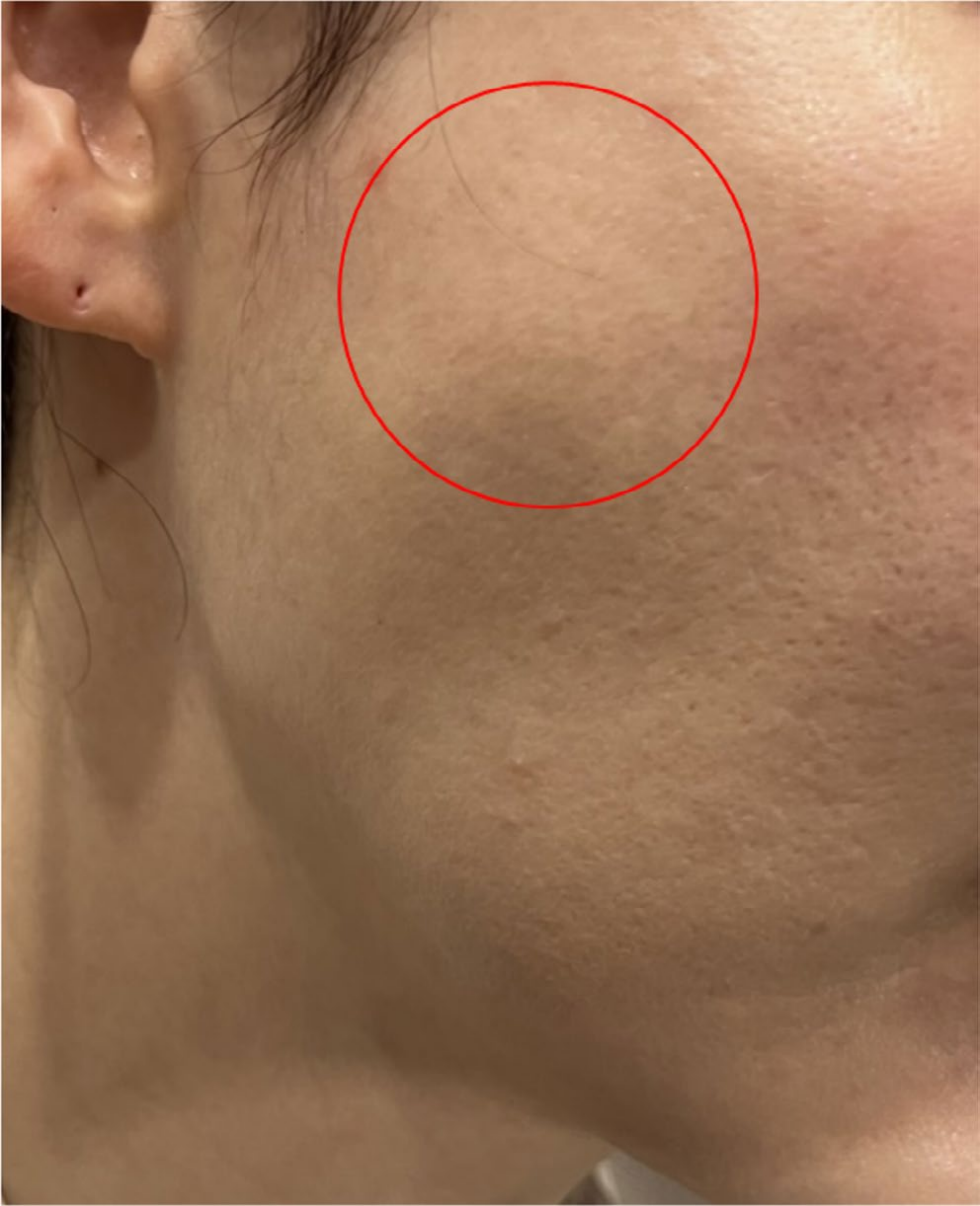
Photograph of a 34‐year‐old female patient presenting with compensatory masseteric bulging. The patient reported a recent asymmetry in facial contour following regular botulinum neurotoxin injections targeting the lower mid‐part of the masseter muscle over 2 years. The image highlights the prominent bulging in the upper portion of the masseter muscle, contributing to the asymmetrical facial appearance.

**FIGURE 2:**
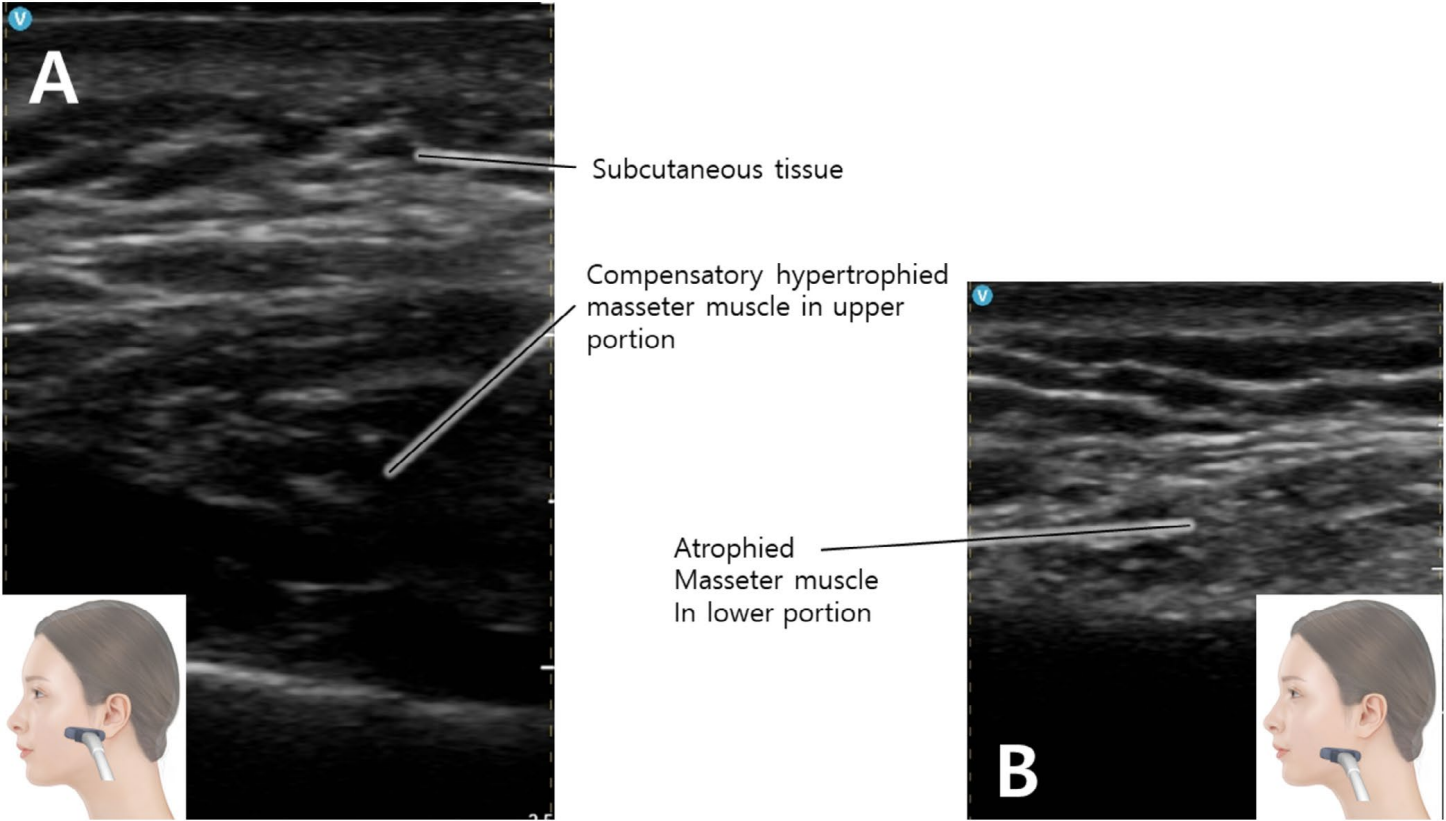
Ultrasound image of the patient's masseter muscle before additional treatment. The image shows a significant difference in thickness between the upper and lower portions of the muscle, with the upper section exhibiting increased bulk with a hypoechoic image at the upper portion (A). This hypertrophy was identified as a compensatory response to repeated botulinum neurotoxin injections in the lower mid‐part of the masseter. The atrophied masseter muscle in the lower portion is observed as a fibrotic hyperechoic image (B).

Clinical examination revealed hypertrophy localized to the upper masseter muscle (Figure 1). Ultrasound imaging further confirmed a significant difference in thickness between the upper and lower portions of the muscle (Figure 2). Given the patient's history of repeated botulinum neurotoxin injections to the lower mid‐part of the masseter, it was concluded that the hypertrophy of the upper masseter muscle was a compensatory response to the localized weakening of the treated area. To address this, 50 units of botulinum neurotoxin (Letibotulinum Toxin A) were injected into the hypertrophied upper portion of the masseter muscle. Follow‐up ultrasound imaging after treatment demonstrated a reduction in the thickness of the upper masseter, indicating a successful response. This case illustrates the phenomenon of compensatory masseteric bulging, highlighting the need for a more comprehensive approach to treatment.

## 4 Discussion

The phenomenon of compensatory masseteric bulging, as identified in this article, presents a significant challenge in the long‐term management of masseter hypertrophy with botulinum neurotoxin, particularly when targeting the lower portion of the masseter for repeated treatments.

This newly recognized complication highlights the need for a more nuanced understanding of the masseter muscle's anatomy and function. The masseter, with its complex three‐layered structure and intricate neural innervation, requires a strategic approach to ensure effective and aesthetically pleasing outcomes without inducing unintended consequences [9].

The occurrence of compensatory hypertrophy in the untreated upper portion of the muscle is particularly concerning due to its proximity to critical anatomical landmarks, including the zygomatic arch and facial nerve branches [10]. Hypertrophy in these areas can lead to asymmetry and other aesthetically undesirable results, which can compromise the overall goals of treatment.

The traditional approach to botulinum neurotoxin injections, which often concentrates on the lower mid‐part of the masseter, may be effective in achieving initial muscle reduction in Asians. In the Asian population, subzygomatic arch depression and an excessively slim jawline are typically not desired aesthetic outcomes. Therefore, treatment strategies in this demographic focus on targeting the lower portion of the masseter to achieve culturally preferred facial contours. Additionally, the study of Kim et al. [11] aimed to determine the intramuscular distribution of the masseteric nerve to identify the most efficient and safe sites for botulinum toxin injection in treating masseteric hypertrophy. Dissections of 12 masseter muscles and Sihler's staining of 10 specimens revealed that the posterosuperior and posteroinferior nerve branches predominantly innervate the deep and middle layers, while anteroinferior branches supply 2–4 perforating twigs to the superficial layer. The richest nerve arborization was localized to Area V, corresponding to the middle and lower portions of the masseter. Based on these findings, the middle and lower portions of the masseter are strongly recommended as the most efficient and safe injection sites for treating masseteric hypertrophy, maximizing therapeutic efficacy and safety.

The conventional injection point is established to prevent an asymmetrical smile, which can occur if botulinum toxin diffuses into adjacent muscles such as the risorius. The study of Bae et al. [12] aimed to investigate the anatomical relationship between the risorius muscle and the masseter muscle to clarify the cause of constrained facial expressions following botulinum neurotoxin Type A injections into the masseter. Dissection of 48 hemifaces revealed that the risorius muscle covers different portions of the masseter, classified into four types: partial coverage of Area III (17.8%), Area VI (20.0%), both Areas III and VI (53.3%), and Areas II, III, and VI (6.7%). The results highlight the medial part of the masseter as a hazard zone, where botulinum neurotoxin injections may inadvertently affect the risorius muscle, potentially causing unnatural facial expressions.

Preventing compensatory masseteric bulging poses a significant challenge; however, when this adverse effect occurs, practitioners should consider carefully administering botulinum neurotoxin to the upper portion of the masseter to mitigate the issue. Patients undergoing botulinum toxin treatment should be informed of the potential for this complication as part of their pre‐treatment counseling.

In conclusion, compensatory masseteric bulging underscores the complexity of treating masseter hypertrophy with botulinum neurotoxin and the need for a holistic approach that considers the entire muscle and its potential responses to treatment. By integrating these considerations into clinical practice, practitioners can improve treatment outcomes and enhance patient satisfaction.

## Author Contributions

K.‐H.Y. and J.W. wrote the main manuscript text. K.‐H.Y. and J.W. prepared Figures 1 and 2. K.‐H.Y. conducted the supervision. All authors reviewed the manuscript.

## Conflicts of Interest

The authors declare no conflicts of interest.

## Data Availability

The data that support the findings of this study are available from the corresponding author upon reasonable request.
